# Supplementary material for: Time Trends in Age at Menarche and Related Non-Communicable Disease Risk during the 20th Century in Mexico
Source: Nutrients. 2019 Feb 13;11(2):394. doi: 10.3390/nu11020394 (PMC6412794; doi:10.3390/nu11020394)
Supplement: Supplementary file 1 [file nutrients-11-00394-s001.pdf]

JUNTOS por la salud

SECRETARIA DE SALUD

SECRETARÍA DE SALUD
Encuesta Nacional de Salud 2000

Cuestionario de Adultos de 20 o más Años de Edad

IDENTIFICACIÓN GEOGRÁFICA

ENTIDAD FEDERATIVA ______________ /___/___/

MUNICIPIO O DELEGACIÓN ______________ /___/___/___/

CLAVE DE AGEB ______________ /___/___/___/ - /___/

LOCALIDAD ______________ /___/___/___/___/

MANZANA ______________ /___/___/___/

CONTROL DE CUESTIONARIO

NÚMERO DE CONTROL .... /___/___/___/___/___/

FOLIO DE VIVIENDA .... /___/___/___/

HOGAR .... /___/ DE /___/

CUESTIONARIO .... /___/ DE /___/

DIRECCIÓN DE LA VIVIENDA (NOMBRE DEL JEFE DE LA FAMILIA)

____

(CALLE, AVENIDA, CALLEJÓN, CARRETERA, CAMINO, BOULEVARD, KM)

NÚMERO EXTERIOR ________ NÚMERO INTERIOR ________ (COLONIA, FRACCIONAMIENTO, BARRIO, UNIDAD HABITACIONAL) ________

RESULTADO DE LA VISITA

NÚMERO DE LA VISITA	1a.	2a.	ÚLTIMA VISITA /___/
NOMBRE Y CLAVE DEL ENTREVISTADOR	/___/___/___/	/___/___/___/	/___/___/___/
HORA DE INICIO	/___/___/ /___/___/	/___/___/ /___/___/	/___/___/ /___/___/
HORA DE TÉRMINO	/___/___/ /___/___/	/___/___/ /___/___/	/___/___/ /___/___/
FECHA (dd mm aaaa)	/__/__/__/__/__/__/__/__/	/__/__/__/__/__/__/__/__/	/__/__/__/__/__/__/__/__/
RESULTADO (*)	/___/	/___/	/___/

(*) CÓDIGO PARA EL RESULTADO DE LA VISITA

1 ENTREVISTA COMPLETA
2 ENTREVISTA INCOMPLETA
3 INFORMANTE INADECUADO
4 AUSENCIA DE OCUPANTES EN EL MOMENTO DE LA VISITA
5 SE NEGÓ A DAR INFORMACIÓN
6 ENTREVISTA APLAZADA (HACER CITA)
7 VIVIENDA DESHABITADA
8 VIVIENDA DE USO TEMPORAL
9 OTRO (ESPECIFIQUE EN OBSERVACIONES)

NÚM. DE REGISTRO DEL ADULTO SELECCIONADO /___/___/

NOMBRE ____

FECHA DE NACIMIENTO /___/___/ /___/___/ /___/___/___/___/
DIA MES AÑO

SEXO /___/
HOMBRE.................... 1
MUJER.................... 2

EDAD /___/___/ /___/___/
AÑOS MESES

ESTADO CONYUGAL
(TRANSCRIBA CLAVE) /___/___/

Ejemplos para las opciones de respuesta de la pregunta 2.2.

**Accidentes de transporte terrestre**: atropellamiento, choque, o caída por automóvil, autobús, tren, bicicleta, carreta, caballo	01
**Otros accidentes de transporte**: accidentes de transporte acuático, Aéreo, teleférico	02
**Caída**: a nivel del piso, desde un mueble, escalera, andamio, árbol, edificio o casa habitación,	03
**Exposición a fuerzas mecánicas inanimadas**: golpe, aplastamiento o contacto traumático con objetos, maquinaria o equipos de elevación, herramientas, vidrios u otros objetos cortantes, disparos, y explosiones accidentales	04
**Exposición a fuerzas mecánicas animadas**: contacto con otras personas, animales o plantas no venenosos	05
**Ahogamiento y sumersión accidentales**: en bañeras, piscinas, aguas naturales	06
**Otros accidentes que obstruyen la respiración**: sofocación o estrangulamiento accidental, sofocación por hundimiento, inhalación de contenido gástrico, alimentos u objetos, encierro en lugar herméticamente cerrado, sofocación por bolsa plástica	07
Exposición a corriente eléctrica, radiación, temperatura y presión del aire **ambientales extremas**: choque eléctrico, exposición a rayos X, rayos UV, láser, calor o frío excesivo artificial, presión atmosférica alta o baja	08
**Exposición al fuego, humo y llamas**: en incendios, fogatas, chimeneas, ignición de combustibles, ropas	09
**Contacto con calor y sustancias calientes**: bebidas. alimentos, agua, vapor, aire caliente, utensilios domésticos, calentadores, motores o metales calientes	10
**Contacto traumático con animales y plantas venenosos**: serpientes arañas, avispas, abejas, ciempiés, medusas	11
**Exposición a fuerzas de la naturaleza**: calor frío natural, radiación solar, rayos, terremoto, avalancha, ciclón, inundación	12
**Envenenamiento accidental**: medicamentos, alcohol, solventes, derivados del petróleo, gases de escape, plaguicidas, raticidas y otros productos químicos	13
**Exposición accidental a otros factores**: exceso de esfuerzo físico, privación de alimentos y agua, y exposición a otros factores	77

SECCIÓN 1. FACTORES DE RIESGO

1.1 ¿Ha fumado usted por lo menos cien cigarrillos (5 cajetillas) de tabaco durante toda su vida?	Sí.................................. 1<br>No.................................. 2<br>Nunca ha fumado.......... 0	/___/<br>(No, Nunca ha fumado) PASE A 1.7
1.2 ¿A qué edad empezó a fumar cigarrillos de tabaco?	No sabe.......................... 88<br>No responde.................. 99	/___/___/<br>años cumplidos
1.3 ¿Actualmente fuma?	Sí..................................... 1<br>No..................................... 2	/___/
1.4 ¿Con qué frecuencia fuma (fumaba)?	Diario.................................. 1<br>Ocasionalmente................. 2	/___/
1.5 Los días que fuma (fumaba) ¿Cuántos cigarrillos consume (consumía)?	No sabe.......................... 88	/___/___/<br>Núm. de cigarrillos
1.6 ¿Durante cuánto tiempo ha fumado (fumó) regularmente?	Menos de un mes .... 0 0<br>No sabe......................... 8 8	meses /__/__/<br>años /__/__/
1.7 ¿A qué edad comenzó a tomar bebidas que contienen alcohol como cerveza, vino, brandy, ron, vodka, tequila, mezcal, pulque, aguardiente o bebidas preparadas con ellos?	Nunca ha tomado....................... 00	/___/___/<br>anote años<br>(Nunca ha tomado) PASE A 1.13
1.8 ¿Actualmente toma?	Sí..................................... 1<br>No..................................... 2	/___/
1.9 ¿Con qué frecuencia toma (o tomaba)?	Diario................................. 1<br>Ocasionalmente................. 2	/___/
1.10 Los días que toma (o tomaba) ¿cuántas copa toma (o tomaba)?	No sabe.......................... 8 8	Núm. de copas<br>/___/___/
1.11 ¿Durante cuánto tiempo ha bebido (o bebió) usted esa cantidad regularmente?	Menos de un mes .... 0 0<br>No sabe......................... 8 8	meses /__/__/<br>años /__/__/

1.12 En el último mes ¿con qué frecuencia tomó lo suficiente como para sentirse borracho o embriagado?	Nunca en el último mes 1<br>Una vez en el mes 2<br>2 o 3 veces en el último mes 3<br>1 o más veces a la semana 4<br>No sabe 8<br>No responde 9	/___/
1.13 ¿Me podría decir si ha tomado, usado o probado por indicación médica o por alguna otra razón?	SÍ NO NO RESPONDE<br>**Opiáceos** **1 2 9**<br>**Tranquilizantes para los nervios** **1 2 9**<br>**Sedantes o barbitúricos para dormir** **1 2 9**<br>**Anfetaminas o estimulantes para adelgazar o dar energía** **1 2 9**<br>**Mariguana** **1 2 9**<br>**Cocaína** **1 2 9**<br>**Heroína** **1 2 9**<br>**Alucinógenos como hongos, peyote, mezcalina, LSD** **1 2 9**<br>**Inhalables como thiner, pegamento, pintura, gasolina** **1 2 9**	/___/<br>/___/<br>/___/<br>/___/<br>/___/<br>/___/<br>/___/<br>/___/<br>/___/

SECCIÓN 2. ACCIDENTES Y VIOLENCIAS

2.1 ¿Ha sufrido algún daño a su salud debido a un accidente en los últimos 12 meses?	Sí.................................. 1<br>No.................................. 2<br>No responde................... 9	/__/<br>(No, No responde) PASE A 2.4
2.2 ¿Cómo fue que se accidentó?<br><br>**REVISE LA LISTA DE EJEMPLOS DE LA PÁGINA DOS PARA SELECCIONAR EL CÓDIGO**	Accidentes de transporte terrestre..................................... 01<br>Otros accidentes de transporte.................................. 02<br>Caída.. 03<br>Exposición a fuerzas mecánicas inanimadas................................ 04<br>Exposición a fuerzas mecánicas animadas.................................. 05<br>Ahogamiento y sumersión accidentales............................... 06<br>Otros accidentes que obstruyen la respiración.................................. 07<br>Exposición a corriente eléctrica, radiación, temperatura............... 08<br>Exposición al fuego, humo y llamas...................................... 09<br>Contacto con calor y sustancias calientes.................................... 10<br>Contacto traumático con animales y plantas venenosos.................. 11<br>Exposición a fuerzas de la naturaleza.................................. 12<br>Envenenamiento accidental........ 13<br>Exposición accidental a otros factores....................................... 77<br>No sabe...................................... 88<br>No responde.............................. 99	/__/__/

2.3 ¿En que lugar se encontraba cuando ocurrió el accidente?	Hogar .... 01<br>Escuela.. 02<br>Trabajo .... 03<br>Vía pública .... 04<br>Campo .... 05<br>Lugar de recreo o deportivo .... 06<br>Establecimiento comercial o de servicios .... 07<br>Otro .... 77<br>No responde 99	/___/___/
2.4 En los últimos 12 meses ¿Sufrió algún daño a su salud por robo, agresión o violencia?	Sí .... 1<br>No .... 2<br>No responde 9	/___/

SI CONTESTÓ SÍ EN 2.1 Y SÍ EN 2.4, CONTINÚE.
SI CONTESTÓ NO EN 2.1 Y NO O NO RESPONDE EN 2.4, PASE A 3.1.
SI CONTESTÓ SÍ EN 2.1 Y NO O NO RESPONDE EN 2.4, PASE A 2.9.
SI CONTESTÓ NO EN 2.1 Y SÍ EN 2.4, CONTINÚE.

2.5 ¿Cuál fue el motivo de la agresión que sufrió?	Robo, asalto .... 01<br>Incidente de tránsito .... 02<br>Agresión sin motivo aparente .... 03<br>Secuestro .... 04<br>Detención .... 05<br>Discusión/altercado .... 06<br>Agresión sexual .... 07<br>Otro .... 77<br>No responde 99	/___/___/
2.6 ¿Cómo o con qué lo agredieron?	Agresión con sustancias .... 01<br>Intento de ahorcamiento o estrangulación .... 02<br>Intento de sofocación o sumersión 03<br>Ataque con arma de fuego y explosivos .... 04<br>Ataque con humo, fuego y llamas. 05<br>Ataque con vapor de agua y objeto caliente .... 06<br>Ataque con arma punzocortante ... 07<br>Ataque con objeto romo (golpe con objeto sin filo) .... 08<br>Empujón desde lugar elevado ...... 09<br>Empujón delante de objeto en movimiento .... 10<br>Colisión con vehículo de motor ..... 11<br>Lucha o pelea sin armas .... 12<br>Ataque sexual .... 13<br>Agresión psicológica .... 14<br>Otro .... 77<br>No responde 99	/___/___/

2.7 ¿En que lugar se encontraba cuando sufrió la agresión?	Hogar 01<br>Escuela 02<br>Trabajo 03<br>Vía pública 04<br>Campo 05<br>Lugar de recreo o deportivo 06<br>Establecimiento comercial o servicios 07<br>Otro 77<br>No responde 99	/___/___/
**EN CASO DE ACCIDENTE Y VIOLENCIA, HAGA LA SIGUIENTE PREGUNTA AL ENTREVISTADO Y REFIÉRASE DE LA 2.9 A 2.20 A ESE EVENTO. SI SÓLO TUVO UNO DE ELLOS, CONTINÚE EN 2.9.**		
2.8 Entre el accidente y la violencia que sufrió ¿Cuál le causo más problemas de salud?	Accidente 1<br>Violencia 2	/___/
2.9 ¿Qué tipo de daño tuvo?<br><br>**ANOTAR OPCIÓN MÁS IMPORTANTE**	Moretón 01<br>Raspón superficial 02<br>Golpe 03<br>Hueso roto 04<br>Hueso o articulación zafado (a) 05<br>Torcedura de articulación 06<br>Cortadura o perforación de la piel o tejido subyacente 07<br>Raspón o rapadura profunda 08<br>Amputación 09<br>Quemadura desde enrojecimiento hasta carbonización 10<br>Intoxicación por sustancia (medicamento, ponzoñas o sustancias químicas) 11<br>Daño psicológico 12<br>Otro 77<br>No sabe 88<br>No responde 99	/___/___/<br><br>(11, 12) **Pase a 2.11**

2.10 ¿Qué parte(s) del cuerpo se lastimó?<br>**PUEDE ANOTAR HASTA 5 OPCIONES, MÁS DE ÉSTAS ANOTE EL CÓDIGO "16"**	Cabeza/cara .... 01<br>Cuello .... 02<br>Tórax .... 03<br>Abdomen .... 04<br>Pelvis .... 05<br>Glúteos .... 06<br>Genitales .... 07<br>Columna vertebral .... 08<br>Hombro .... 09<br>Brazo(s) .... 10<br>Antebrazo (s) .... 11<br>Mano (s) .... 12<br>Muslo (s) .... 13<br>Pierna (s) .... 14<br>Pie (s) .... 15<br>Todo el cuerpo .... 16<br>Otro .... 77<br>No sabe .... 88<br>No responde 99	/__/__/<br>/__/__/<br>/__/__/<br>/__/__/<br>/__/__/
2.11 ¿Cuándo le ocurrió el accidente o violencia?	Últimas 2 semanas .... 1<br>De 3 semanas a 2 meses .... 2<br>De 3 a 6 meses .... 3<br>De 7 a 12 meses .... 4<br>No responde 9	/__/
2.12 ¿Cuántos días dejó de estudiar, trabajar o hacer sus actividades de la vida diaria por este problema?	Ningún día .... 000<br>No responde 999	/__/__/<br>Anote días
2.13 ¿Actualmente tiene problemas de salud ocasionados por este accidente o violencia?	Sí .... 1<br>No .... 2<br>No responde 9	/__/<br>(No, No responde) **PASE A 2.15**
2.14 ¿Qué tipo de problema de salud le ocasionó este accidente o violencia?	Limitación o dificultad para moverse o caminar (o lo hace con ayuda) ... 1<br>Limitación o dificultad para usar sus brazos y manos .... 2<br>Sordera o dificultad para oír .... 3<br>Pérdida de la función vocal (mudo) 4<br>Dificultad para ver o ceguera .... 5<br>Retraso o deficiencia mental .... 6<br>Tiene otra limitación física o mental<br>____________ 7<br>Especifique<br>No sabe .... 8<br>No responde 9	/__/

2.15 ¿Qué hizo o quién lo atendió?	Nada o nadie .... 01<br>Remedios caseros, automedicación .... 02<br>Curandero(a) o yerbero(a) .... 03<br>Huesero(a) o sobador(a) .... 04<br>Encargado(a) comunidad .... 05<br>Psicólogo, terapeuta .... 06<br>Médico, consultorio .... 07<br>Clínica, sanatorio u hospital .... 08<br>Otro .... 77<br>No responde 99	/___/___/<br>PASE A 2.17
2.16 ¿Por qué no solicitó atención médica por lo que le pasó?	No fue necesario .... 01<br>Es caro .... 02<br>Está muy lejos .... 03<br>Falta de confianza .... 04<br>Tratan mal .... 05<br>Falta de tiempo .... 06<br>No hay clínica/hospital .... 07<br>Otro .... 77<br>No responde 99	/___//___/
2.17 ¿Cuando sufrió el accidente o violencia, estaba bajo los efectos de...	**alcohol?** .... **1**<br>**drogas?** .... **2**<br>**otro?**___________<br>Especifique<br>No estaba bajo efectos .... 0<br>No responde 9	/___/

SÓLO EN CASO DE ACCIDENTE O VIOLENCIA INFRINGIDO POR TERCERA PERSONA; SI NO ES ASÍ, PASE A 3.1.

2.18 ¿La persona que lo lastimó estaba bajo los efectos de...	**alcohol?** .... **1**<br>**drogas?** .... **2**<br>**otro?**___________<br>Especifique<br>No estaba bajo efectos .... 0<br>No sabe .... 8<br>No responde 9	/___/
2.19 ¿Conocía a la persona que lo lastimó?	Sí .... 1<br>No .... 2<br>Inseguro .... 3<br>No responde 9	/___/<br>PASE A 3.1
2.20 ¿Quién fue la persona que lo lastimó?	Pareja .... 1<br>Familiar .... 2<br>Amigo .... 3<br>Vecino u otro conocido .... 4<br>No responde 9	/__/

SECCIÓN 3. ANTECEDENTES HEREDO-FAMILIARES

3.1 ¿Tienen (o tenían) sus padres algún parentesco entre sí?	Sí .... 1<br>No .... 2<br>No sabe .... 8<br>No responde 9	/__/

De los siguientes familiares a, su_____________ algún médico le ha dicho que tiene o tuvo _____________
Familiar Enfermedad

FAMILIAR (ES)	3.2 ...diabetes (o alta el azúcar en la sangre)?			3.3 ...la presión alta?			3.4 ...problemas del corazón?		
	Sí	No	No sabe	Sí	No	No sabe	Sí	No	No sabe
a) Padre........	1	2	/__/ 8	1	2	/__/ 8	1	2	/__/ 8
b) Madre........	1	2	/__/ 8	1	2	/__/ 8	1	2	/__/ 8
c) Hermano (a) gemelo (a)........ ¿cuántos?	/__/__/	22	88	/__/__/	22	88	/__/__/	22	88
d) Hermanos (as)........ ¿cuántos?	/__/__/	22	88	/__/__/	22	88	/__/__/	22	88
e) Abuelos (as)........ ¿cuántos?	/__/__/	22	88	/__/__/	22	88	/__/__/	22	88
f) Tíos (as)........ ¿cuántos?	/__/__/	22	88	/__/__/	22	88	/__/__/	22	88
g) Hijos (as)........ ¿cuántos?	/__/__/	22	88	/__/__/	22	88	/__/__/	22	88

SECCION 4. SALUD REPRODUCTIVA

AHORA LE VOY HACER PREGUNTAS SOBRE MÉTODOS PARA NO TENER HIJOS (EMBARAZOS, MENSTRUACIÓN) Y ALGUNAS SOBRE CIERTAS ENFERMEDADES QUE PUDO HABER PRESENTADO DURANTE LOS ÚLTIMOS 12 MESES.

4.1 ¿A qué edad tuvo usted su primera relación sexual?	No ha tenido relaciones sexuales. 00<br>No responde .... 99<br>No sabe o no recuerda 88	/__/__/<br>**anote años**<br>**MUJERES 4.18**<br>**HOMBRES 5.1**
**SÓLO PARA MUJERES (HOMBRES, PASE A 4.13)**		
4.2 ¿Ha estado usted embarazada alguna vez?<br>**NO CONSIDERE EMBARAZO ACTUAL, CUANDO ESTE SEA EL PRIMERO**	Sí .... 1<br>No .... 2<br>No sabe .... 8<br>No responde 9	/__/<br>**PASE A 4.11**
4.3 ¿De estos embarazos cuántos han:<br>**ANOTE EMBARAZOS SEGÚN PRODUCTO**	**nacido vivos, aunque hayan muerto poco tiempo después?..................**<br>**nacido muertos?..........................**	**número**<br>/__/__/<br>/__/__/
4.4 ¿Cuántas pérdidas o abortos ha tenido?	No responde 99	/__/__/<br>**número**
**SÓLO PARA MUJERES CON HIJOS NACIDOS VIVOS 4.3; SI NO, PASE A 4.11**		
4.5 ¿En qué mes y año nació su último(a) hijo(a) nacido vivo?	No recuerda 88 8888	/_/_/<br>**mes**<br>/__/__/__/__/<br>**año**
**SÓLO PARA MUJERES CUYO ÚLTIMO HIJO NACIÓ VIVO DE 1994 A LA FECHA; SI NO, PASE A 4.11.**		
4.6 Cuando estuvo embarazada de su último hijo ¿Quién y cuántas veces la revisó por el embarazo?<br>**ANOTE HASTA DOS OPCIONES**	Médico .... 1<br>Enfermera .... 2<br>Promotora, auxiliar o asistente de salud .... 3<br>Partera .... 4<br>Otro personal .... 7<br>Nadie .... 0<br>No responde 9	***Persona*** ***veces***<br>/__/ /_/_/<br>/__/ /_/_/<br>**PASE A 4.8**

4.7 ¿Cuántos meses de embarazo tenía cuando la revisaron por primera vez?	No recuerda 88	/__/__/ meses
4.8 ¿Quién la atendió de su último parto?	Médico .... 1<br>Enfermera .... 2<br>Promotora, auxiliar o asistente de salud .... 3<br>Partera .... 4<br>Pariente o familiar .... 5<br>Otro personal .... 7<br>Nadie .... 0<br>No responde 9	/__/<br>(0, 9) PASE A 4.11
4.9 ¿En dónde la atendieron de su último parto?	Seguro Social (clínica u hospital) .... 01<br>ISSSTE (clínica u hospital) .... 02<br>SSA (centro de salud u hospital) .... 03<br>IMSS Sol. (clínica u hospital) .... 04<br>Otra institución de salud del gobierno .. 05<br>Consultorio, clínica u hospital privado .. 06<br>Casa de la partera .... 07<br>Casa de la entrevistada .... 08<br>Otro lugar 77	/__/__/
4.10 Su último parto fue...	**normal?** .... 1<br>**cesárea?** 2	/__/
**SI LA MUJER QUE ESTA ENTREVISTANDO ES MAYOR DE 49 AÑOS, PASE A 4.18**		
4.11 ¿Está usted embarazada actualmente?	Sí .... 1<br>No .... 2<br>No sabe 8	/__/
**SI CONTESTÓ SÍ EN 4.2 O EN 4.11, PASE A 4.13**		
4.12 ¿Cuál es la razón por la que no se ha embarazado?	No desea embarazarse .... 1<br>Le han diagnosticado esterilidad .... 2<br>Le han diagnosticado esterilidad a su esposo .... 3<br>Otra .... 4<br>No sabe .... 8<br>No responde 9	/__/<br>(2, 3) PASE A 4.18

PARA HOMBRES Y MUJERES DE 20 A 49 AÑOS DE EDAD;
SI NO ES ASÍ, HOMBRES PASE A 4.22 Y MUJERES A 4.18

Pregunta	Respuesta	Código	Pase	Registro
4.13 Actualmente ¿usted o su pareja están haciendo algo para no tener hijos?	Sí	1		/__/
	No	2	PASE A 4.15	
	No tiene pareja	3	HOMBRE A 4.22 MUJER A 4.18	
	No responde	9		
4.14 ¿Están usted o su pareja operados para ya no tener hijos?	Sí **¿Quién?**			/__/
	Ella (o él)	1	PASE A 4.16	
	Su pareja	2		
	No	3	PASE A 4.17	
	No responde	4	HOMBRE A 4.22 MUJER A 4.18	
4.15 ¿Qué están actualmente haciendo usted o su pareja para no tener hijos? **PUEDE ANOTAR MÁS DE UNA OPCIÓN**	Operación femenina o ligadura	01		/__/__/
	Operación masculina o vasectomía	02		/__/__/
	Pastillas o píldoras	03		/__/__/
	Inyecciones	04		/__/__/
	Norplant	05		/__/__/
	Dispositivo, DIU	06		/__/__/
	Preservativo o condón	07		
	Óvulos, jaleas o espumas	08		
	Ritmo, calendario, abstinencia periódica, termómetro, Billings	09	HOMBRE A 4.22 MUJER A 4.18	
	Retiro o coito interrumpido	10		
	Otro	77		
	No responde	99		

REVISE LAS RESPUESTAS DE LA PREGUNTA ANTERIOR Y REALICE LA PREGUNTA 4.16 SÓLO PARA EL MÉTODO QUE TENGA EL CÓDIGO MENOR

Pregunta	Respuesta	Código	Pase	Registro
4.16 ¿Dónde consiguió (le pusieron o la operaron) _____________ método	Seguro Social (clínica u hospital)	01	HOMBRE A 4.22 MUJER A 4.18	/__/__/
	ISSSTE (clínica u hospital)	02		
	SSA (centro de salud u hospital)	03		
	IMSS Sol. (clínica u hospital)	04		
	DIF	05		
	Otra institución de salud del gobierno	06		
	MEXFAM	07		
	FEMAP	08		
	Consultorio, clínica u hospital privado	09		
	Farmacia	10		
	Tienda de autoservicio	11		
	Otro lugar	77		
	No sabe	88		

4.17 ¿Cuál es la razón principal por la que actualmente no están haciendo algo para no tener hijos?	Está embarazada .... 01<br>Quiere embarazarse .... 02<br>No lo necesita. Es soltera, separada, viuda o divorciada .... 03<br>Por esterilidad de ella o su pareja .. 04<br>Por ausencia temporal de su pareja .... 05<br>Porque dejó de tener relaciones sexuales .... 06<br>Teme a los efectos colaterales ...... 07<br>Tuvo efectos colaterales .... 08<br>Se opone su pareja .... 09<br>Tiene alguna enfermedad y prefiere no usar nada .... 10<br>Está dando el pecho y no considera necesario usar método .... 11<br>Y cree que puede haber problemas de salud .... 12<br>Por razones religiosas .... 13<br>No ésta de acuerdo en usar métodos .... 14<br>No conoce métodos .... 15<br>No sabe cómo se usan los métodos .... 16<br>Otra razón .... 77<br>No sabe .... 88<br>No responde 99	/__/__/<br>**HOMBRES A 4.22**
4.18 ¿A qué edad tuvo su primera regla o menstruación?	No ha comenzado .... 00<br>No sabe .... 88<br>No responde 99	**PASE A 4.22**<br>/__/__/<br>**anote edad**
4.19 ¿Cuándo fue su última regla o menstruación?	Menos de un mes .... 96<br>Actualmente está reglando .... 97<br>No sabe 88	/__/__/<br>**mes**<br>/__/__/__/__/<br>**año**
**FILTRO**	**ÚLTIMA MENSTRUACIÓN HACE MENOS DE 3 MESES, PASE A PREGUNTA 4.22**	

4.20 ¿Cuál fue la razón por la que usted dejó de reglar?	Está amamantando o en puerperio 01<br>Actualmente está embarazada .... 02<br>Por menopausia natural .... 03<br>Le quitaron la matriz o los ovarios .. 04<br>Recibió radiaciones en la pelvis ...... 05<br>Tomó medicamentos o quimioterapia .... 06<br>Otras razones .... 77<br>No sabe .... 88<br>No responde 99	/___/___/<br>**PASE A 4.22** (01, 02)
4.21 ¿Cuántos años cumplidos tenía usted cuando dejó de reglar?	No sabe .... 88<br>No responde 99	/___/___/<br>**años**
**SÓLO PARA QUIENES EN 4.1 REFIRIERON HABER TENIDO RELACIONES SEXUALES**		
4.22 En los últimos 12 meses ha tenido alguna de las siguientes enfermedades<br><br>**PUEDE ANOTAR MÁS DE UNA OPCIÓN**	**verrugas genitales?** .... **1**<br>**gonorrea?** .... **2**<br>**sífilis?** .... **3**<br>**otras enfermedades de transmisión sexual**<br>_______________ .... **7**<br>**Especifique**<br>Ninguna .... 0<br>No sabe .... 8<br>No responde 9	/___/<br>/___/<br>/___/<br>/___/<br>/___/<br>**PASE A 5.1** (0, 8, 9)
4.23 ¿Qué hizó cuando se enfermo de (mencione la última enfermedad señalada en pregunta anterior)?<br><br>**PUEDE ANOTAR MÁS DE UNA OPCIÓN**	**¿Lo consultó con amigos(a)** .... **01**<br>**¿Usó remedios caseros?** .... **02**<br>**¿Usó medicamentos que tenía en casa o que anunciaban por televisión?** .... **03**<br>**¿Consultó a curanderos (a) o médico tradicional?** .... **04**<br>**¿Fue a la clínica/centro de salud o con promotor (a) o asistente de salud?** .... **05**<br>**¿Consultó un médico privado?** ... **06**<br>**¿Consultó al farmacéutico?** .... **07**<br>**¿Otro?** .... **08**<br>No hizo nada .... 00<br>No responde 99	/___/___/<br>/___/___/<br>/___/___/<br>/___/___/<br>/___/___/<br>/___/___/

SECCIÓN 5. DIABETES MELLITUS

5.1 ¿Actualmente tiene alguna de estas molestias...<br><br>**PUEDE ANOTAR MÁS DE UNA OPCIÓN**	**Sí No**<br>**mucha sed? 1 2**<br>**orina mucho? 1 2**<br>**mucha hambre? 1 2**<br>**pérdida de peso? 1 2**<br>**visión borrosa? 1 2**	/___/<br>/___/<br>/___/<br>/___/<br>/___/
5.2 ¿Algún médico le ha dicho que tiene diabetes o alta el azúcar en la sangre?	Sí 1<br>No 2	/___/<br>**PASE A 6.1**
5.3 ¿Hace cuánto tiempo le dijo su médico por primera vez que tenía diabetes o alta el azúcar en la sangre?	Menos de un mes 00<br>No sabe 88	**meses** /___/___/<br>**años**/___/___/
5.4 ¿Cómo diagnosticó el médico que tenía diabetes o alta el azúcar en la sangre?<br><br>**PUEDE ANOTAR MÁS DE UNA OPCIÓN**	Un examen de sangre 1<br>Un examen de orina 2<br>Por los síntomas que presentaba (molestias) 3<br>Otras 7	/___/<br>/___/<br>/___/<br>/___/
5.5 ¿Ha tenido tratamiento médico para controlar su azúcar en la sangre?	Sí 1<br>No 2	/___/<br>**PASE A 5.10**
5.6 ¿En dónde se atiende para controlar su diabetes?	IMSS Solidaridad 01<br>IMSS 02<br>SSA 03<br>DIF 04<br>DDF 05<br>INI 06<br>ISSSTE estatal 07<br>ISSSTE 08<br>Marina/Defensa 09<br>PEMEX 10<br>Particular 11<br>Cruz Roja 12<br>SEP 13<br>ONG 14<br>Otro 77<br>No sabe 88<br>No responde 99	/___/___/

5.7 ¿Actualmente toma pastillas o le aplican insulina para controlar su azúcar?	Ninguno 0<br>Pastillas 1<br>Insulina 2	PASE A 5.10<br><br>/__/
5.8 ¿Me puede mostrar el medicamento que está tomando?	Lo mostró 1<br>No lo mostró 2	/__/
5.9 ¿Cuánto tiempo tiene con este tratamiento?	Menos de un mes 00<br>No sabe 88	**meses** /__/__/<br>**años** /__/__/
5.10 ¿Actualmente lleva algún otro tratamiento para controlar su azúcar?<br><br>**PUEDE ANOTAR MÁS DE UNA OPCIÓN**	Ninguno 0<br>Plan de alimentación 1<br>Realiza algún ejercicio físico 2<br>Homeopatía (chochos) 3<br>Herbolaria 4<br>Hemoterapia 5<br>Otros 7	/__/<br>/__/<br>/__/<br>/__/<br>/__/<br>/__/<br>/__/
5.11 ¿Qué exámenes *se hace* o le ordena su médico para vigilar su azúcar?<br><br>**PUEDE ANOTAR MÁS DE UNA OPCIÓN**	Ninguno 0<br>Tiras reactivas en orina 1<br>Tiras reactivas en sangre 2<br>Examen general de orina 3<br>Determinación de glucosa en sangre venosa 4<br>Control de fructosamina 5<br>Determinación de hemoglobina glucosilada 6<br>Otro 7	/__/**PASE A 6.1**<br>/__/<br>/__/<br>/__/<br>/__/<br>/__/<br>/__/<br>/__/
5.12 ¿Con qué frecuencia se realiza los exámenes para conocer el valor de su azúcar?	Días...<br>Meses..<br>Años...<br>No sabe 88	/__//__/<br>/__//__/<br>/__//__/

SECCIÓN 6. HIPERTENSIÓN ARTERIAL

6.1 ¿Actualmente tiene alguna de estas molestias...	**Sí No**<br>**dolor de cabeza? 1 2**<br>**mareos? 1 2**<br>**zumbido en los oídos? 1 2**<br>**ha visto destellos de luz sin ninguna molestia? 1 2**<br>**sangrado de la nariz sin ningún motivo? 1 2**	/___/<br>/___/<br>/___/<br>/___/<br>/___/
6.2 ¿Alguna vez le han tomado la presión arterial?	Sí 1<br>No 2	/___/<br>**PASE A 7.1**
6.3 ¿Algún médico le ha dicho que tiene la presión alta?	Sí 1<br>No 2	/___/<br>**PASE A 7.1**
6. 4 ¿Hace cuánto tiempo le dijo su médico que tiene la presión alta?	Menos de un mes 00<br>No sabe 88	**meses** /___/___/<br>**años**/___/___/
6.5 ¿Ha tenido tratamiento médico para controlar su presión alta?	Sí 1<br>No 2	/___/<br>**PASE A 6.10**
6.6 ¿En dónde se atiende para controlar su presión alta?	IMSS Solidaridad 01<br>IMSS 02<br>SSA 03<br>DIF 04<br>DDF 05<br>INI 06<br>ISSSTE estatal 07<br>ISSSTE 08<br>Marina/Defensa 09<br>PEMEX 10<br>Particular 11<br>Cruz Roja 12<br>SEP 13<br>ONG 14<br>Otro 77<br>No sabe 88<br>No responde 99	/___/___/

6.7 ¿Actualmente toma algún medicamento para controlar su presión alta?	Sí .... 1<br>No 2	/___/<br>PASE A 6.10
6.8 ¿Me puede mostrar el medicamento que esta tomando?	Lo mostró .... 1<br>No lo mostró 2	/___/
6.9 ¿Cuánto tiempo tiene tomando este medicamento?	Menos de un mes .... 00<br>No sabe 88	**meses** /___/___/<br>**años** /___/___/
6.10 ¿Actualmente lleva algún otro tratamiento para controlar su presión alta?<br><br>**PUEDE ANOTAR MÁS DE UNA OPCIÓN**	Ninguno .... 0<br>Plan de alimentación .... 1<br>Realiza algún ejercicio físico .... 2<br>Homeopatía (chochos) .... 3<br>Herbolaria .... 4<br>Disminución en el consumo de sal 5<br>Otros 7	/___/ **PASE A 7.1**<br>/___/<br>/___/<br>/___/<br>/___/<br>/___/<br>/___/
6.11 ¿Cada cuándo se toma la presión arterial?	Días........<br>Meses........<br>Años........<br>No se la toman .... 00<br>No sabe 88	/___/<br>/___/<br>/___/<br>/___/ **PASE A 7.1**<br>/___/
6.12 ¿Qué persona le toma la presión arterial fuera del consultorio?	Médico .... 1<br>Enfermera .... 2<br>Usted mismo .... 3<br>Auxiliar no personal de salud .... 4<br>Siempre se la toman en el consultorio 5	/___/

SECCIÓN 7. ENFERMEDAD RENAL Y ENFERMEDADES REUMÁTICAS

Pregunta	Opciones	Sí	No	Código
7.1 En las últimas dos semanas ¿ha presentado alguna de las siguientes molestias...	se le han hinchado los pies, manos o cara?	1	2	/__/
	ha expulsado piedras en la orina?	1	2	/__/
	orina muy a menudo (poliuria)?	1	2	/__/
	su orina es muy escasa (oliguria)?	1	2	/__/
	tiene retención de orina?	1	2	/__/
	ardor o dolor al orinar?	1	2	/__/
	pujo al orinar?	1	2	/__/
	dolor en el riñón?	1	2	/__/
7.2 ¿Algún médico le ha dicho que padece alguna de las siguientes enfermedades...	infección de vías urinarias?	1	2	/__/
	cálculos renales?	1	2	/__/
	insuficiencia renal?	1	2	/__/
	alguna enfermedad de la próstata (sólo hombres)?	1	2	/__/ PASE A 7.4

SI ALGUNA DE LAS RESPUESTAS DE LA PREGUNTA ANTERIOR CORRESPONDE EL CÓDIGO 1, CONTINÚE; SI NO ES ASÍ, PASE A 7.4

Pregunta	Opciones	Código	
7.3 ¿Actualmente que tratamiento tiene para su enfermedad renal? **PUEDE ANOTAR MÁS DE UNA OPCIÓN**	Ninguno	0	/__/
	Dieta sin sal	1	/__/
	Medicamentos	2	/__/
	Diálisis	3	/__/
	Homeopático	4	/__/
	Acupuntura	5	/__/
	Naturista	6	/__/
	Otro	7	/__/

Pregunta	Opciones	Sí	No	Código
7.4 ¿En las últimas dos semanas ha tenido inflamación o dolor en alguna de las siguientes articulaciones o coyunturas...	del dedo gordo del pie?	1	2	/__/
	del pie o talón?	1	2	/__/
	de las rodillas?	1	2	/__/
	de los codos?	1	2	/__/
	de los hombros?	1	2	/__/
	de las manos o muñecas?	1	2	/__/
	de la cadera?	1	2	/__/

7.5 ¿Algún médico le ha dicho que padece (o padeció) alguna de las siguientes enfermedades reumáticas...		**Sí**	**No**	
	**gota (ácido úrico alto)?**	**1**	**2**	/__/
	**artritis por gota?**	**1**	**2**	/__/
	**artritis reumatoide?**	**1**	**2**	/__/
	**otras artritis?**	**1**	**2**	/__/
	**otra enfermedad causante de inflamación o dolor?**	**1**	**2**	/__/
**SI ALGUNA DE LAS RESPUESTAS DE LA PREGUNTA ANTERIOR CORRESPONDE EL CÓDIGO 1, CONTINÚE; SI NO, PASE A 7.7**				
7.6 ¿Ha tomado algún medicamento para controlar su... **VEA RESPUESTAS DE PREGUNTA ANTERIOR**		**Sí**	**No**	
	**gota (ácido úrico alto)?**	**1**	**2**	/__/
	**artritis por gota?**	**1**	**2**	/__/
	**artritis reumatoide?**	**1**	**2**	/__/
	**otras artritis?**	**1**	**2**	/__/
	**otra enfermedad causante de inflamación o dolor?**	**1**	**2**	/__/
7.7 ¿Algún médico le ha dicho que tiene el colesterol alto?	Sí	1		/__/
	No	2		
7.8 ¿En general considera que su estado de salud en el último año ha sido.....	**bueno?**	**1**		/__/
	**regular?**	**2**		
	**malo?**	**3**		
	No sabe	8		
	No responde	9		

SECCIÓN 8. PROGRAMAS PREVENTIVOS

8.1 ¿Durante los últimos 12 meses acudió al módulo de medicina preventiva para...<br><br>Sí......... 1<br>No....... 2 **pase a la siguiente prueba de detección**<br><br>**SI EL INFORMANTE ES HOMBRE, INICIE EN EL INCISO C**	8.2 ¿En qué institución le dieron el servicio?<br><br>IMSS Solidaridad .... 01<br>IMSS .... 02<br>SSA .... 03<br>DIF .... 04<br>DDF .... 05<br>INI .... 06<br>ISSSTE estatal .... 07<br>ISSSTE .... 08<br>Marina/Defensa .... 09<br>PEMEX .... 10<br>Particular .... 11<br>Cruz Roja .... 12<br>SEP .... 13<br>ONG .... 14<br>Otro .... 77<br>No sabe .... 88<br>No responde 99	8.3 ¿Presentaba alguna molestia por la cual le realizaron la prueba de detección?<br><br>Sí............ 1<br>No........... 2	8.4 ¿Le entregaron el resultado del estudio?<br><br>Sí............ 1<br>No........... 2<br><br>**PASE A SIGUIENTE PRUEBA DE DETECCIÓN O SECCIÓN**
/__/<br>a) Prueba de Papanicolaou	/__/__/	/__/	/__/
/__/<br>b) Detección de cáncer de mama (exploración clínica)	/__/__/	/__/	/__/
/__/<br>c) Detección de tuberculosis (análisis de flema o esputo)	/__/__/	/__/	/__/
/__/<br>d) Detección de diabetes (prueba del azúcar)	/__/__/	/__/	/__/
/__/<br>e) Detección de hipertensión (toma de presión arterial)	/__/__/	/__/	/__/

	8.5 ¿Qué diagnóstico le dieron?<br><br>Positivo .... 1<br>Negativo .... 2<br>No sabe .... 8<br>No responde .... 9<br><br>**PASE A SIGUIENTE PRUEBA DE DETECCIÓN O SECCIÓN**	8.6 ¿Recibió tratamiento?<br><br>Sí........... 1<br>No........... 2<br><br>**PASE A SIGUIENTE PRUEBA DE DETECCIÓN O SECCIÓN**	8.7 ¿Cuál es la causa por la que no recibió tratamiento?<br><br>No hay donde atenderse .... 01<br>Es caro .... 02<br>No tenía dinero 03<br>La unidad médica le queda lejos ..... 04<br>Falta de confianza .... 05<br>Tratan mal ...... 06<br>No tuvo tiempo 07<br>Fue pero no lo atendieron ..... 08<br>Otro .... 77<br>No sabe .... 88<br>No responde ... 99
a) Prueba de Papanicolaou	/___/___/	/___/	/___/___/
b) Detección de cáncer de mama (exploración clínica)	/___/___/	/___/	/___/___/
c) Detección de tuberculosis (análisis de flema o esputo)	/___/___/	/___/	/___/___/
d) Detección de diabetes (prueba del azúcar)	/___/___/	/___/	/___/___/
e) Detección de hipertensión (toma de presión arterial)	/___/___/	/___/	/___/___/

SECCIÓN 9. HOJA DE REGISTRO DE MEDICIONES CLÍNICAS

9.1 ¿Se realizó la medición de peso?	Sí........... 1 /___/___/___/ /___/___/___/Kg<br>No.......... 2 ¿Por qué? ______________<br>Anote causa	/___/
9.2 ¿Se realizó la medición de talla de pie?	Sí........... 1 /___/___/___/ /___/ cm<br>No.......... 2 ¿Por qué? ______________<br>Anote causa	/___/
9.3 ¿Se realizó la medición de cintura?	Sí........... 1 /___/___/___/ /___/ cm<br>No.......... 2 ¿Por qué? ______________<br>Anote causa	/___/
9.4 ¿Se realizó la primera toma de tensión arterial?	Sí........... 1 /___/___/___//___/___/___/<br>TAS TAD<br>No.......... 2 ¿Por qué? ______________<br>Anote causa	/___/
**ESPERE CUANDO MENOS 5 MINUTOS ANTES DE REALIZAR LA SEGUNDA TOMA DE TENSIÓN ARTERIAL**		
9.5 ¿Se realizó la segunda toma de tensión arterial?	Sí......... 1 /___/___/___/ /___/___/___/<br>TAS TAD<br>No........ 2 ¿Por qué? ______________<br>Anote causa	/___/

SECCIÓN 10. HOJA DE REGISTRO DE MEDICIONES BIOLÓGICAS

10.1 ¿Ha comido o tomado algún alimento entre las últimas 8 a 12 horas?	Sí.. 1<br>No.. 2	/__/
10.2 ¿Se tomó muestra de sangre capilar para glucemia?	Sí.. 1<br>No.. 2 →	/__/<br>**PASE A 10.4**
10.3 Resultado de la glucemia	____________mg/dl	/__/
10.4 ¿Se tomó muestra de sangre venosa en tubo con SST?	No........... 2 Anote causa ___________<br>Sí............. 1<br><br>Fecha I__I__I I__I__I I__I__I__I__I<br>día mes año	/__/
10.5 ¿Tomó muestra de sangre venosa en tubo con EDTA?	No........... 2 Anote causa ___________<br>Sí............. 1<br><br>Fecha I__I__I I__I__I I__I__I__I__I<br>día mes año	/__/
**En este espacio coloque la etiqueta de control**		
10.6 ¿Se tomó muestra de orina?	Sí.................. 1<br>No................ 2 Anote causa ________	/__/
10.7 Resultado del examen general de orina con tira reactiva.	Peso específico .... /________/<br>**PH** .... /________/<br>**Leucocitos** .... /________/<br>**Nitritos** .... /________/<br>**Proteínas** .... /________/<br>**Glucosa** .... /________/<br>**Cetonas** .... /________/<br>**Urobilinógeno** .... /________/<br>**Bilirrubina** .... /________/<br>**Sangre** .... /________/<br>**Hemoglobina**........................... /________/	

EN CASO DE OBTENER EXAMEN NEGATIVO O TRAZAS EN PROTEÍNAS (PRO), REALIZAR EXAMEN CON MICRAL-TEST.

10.8 Resultado del examen con Micral-test	0 mg/l..................................... 1<br>20 mg/l.................................. 2<br>50 mg/l.................................. 3<br>100 mg/l................................. 4	/__/

RESPETO A LAS PERSONAS

De acuerdo con el Artículo 13, párrafo primero, del Reglamento de la Ley General de Salud en materia de Investigación para la Salud, en vigor: "En toda investigación en la que el ser humano sea sujeto de estudio, deberá prevalecer el criterio del respeto a su dignidad y la protección a sus derechos y su bienestar".

OBLIGATORIEDAD

De acuerdo con el **Artículo 42, párrafo primero, de la Ley de Información Estadística y Geografía**, en vigor: "Los informantes estarán obligados a proporcionar con veracidad y oportunidad los datos e informes que les solicitan las autoridades competentes para fines estadísticos, censales y geográficos y a prestar el auxilio y cooperación que requieran las mismas".

CONFIDENCIALIDAD

Conforme a las disposiciones del **Artículo 16, del Reglamento de la Ley General de Salud en materia de Investigación para la Salud**, en vigor; "En las investigaciones en seres humanos se protegerá la privacidad del individuo sujeto de investigación, identificándolo sólo cuando los resultados lo requieran y este lo autorice".

En referencia directa el **Artículo 38, de la Ley de Información Estadística y Geográfica**, en vigor, enuncia: "Los datos e informes que los participantes proporcionen para fines estadísticos o provengan de registros administrativos o civiles, serán manejados para efectos de esta Ley, bajo la observancia de los principios de confidencialidad y reserva y no podrán comunicarse, en ningún caso, en forma nominativa o individualizada, ni harán prueba ante autoridad administrativa o fiscal, ni en juicio o fuera de él".

OBSERVACIONES

____

____

____

____

____

____

____

____

____

____

____

____

____

RECUADRO DE CONTROL

	SUPERVISOR	*CODIFICACIÓN*	*CAPTURA*
Nombre:			
Clave:	/__/__/__/	/__/__/__/	/__/__/__/
Fecha:	/_/_//_/_//_/_/_/_/	/_/_//_/_//_/_/_/_/	/_/_//_/_//_/_/_/_/
